# Functional and structural brain connectivity of young binge drinkers: a follow-up study

**DOI:** 10.1038/srep31293

**Published:** 2016-08-10

**Authors:** A. Correas, P. Cuesta, E. López-Caneda, S. Rodríguez Holguín, L. M. García-Moreno, J. A. Pineda-Pardo, F. Cadaveira, F. Maestú

**Affiliations:** 1 Laboratory of Cognitive and Computational Neuroscience, Centre of Biomedical Technology (CTB), 28223, Madrid, Spain; 2 Neuropsychophysiology Lab, Research Center on Psychology (CIPsi), School of Psychology, 4710, University of Minho, Braga, Portugal; 3 Department of Clinical Psychology and Psychobiology, University of Santiago de Compostela, 15782, Santiago de Compostela, Spain; 4 Department of Psychobiology, Complutense University Madrid, 28040, Madrid, Spain

## Abstract

Adolescence is a period of ongoing brain maturation characterized by hierarchical changes in the functional and structural networks. For this reason, the young brain is particularly vulnerable to the toxic effects of alcohol. Nowadays, binge drinking is a pattern of alcohol consumption increasingly prevalent among adolescents. The aim of the present study is to evaluate the evolution of the functional and anatomical connectivity of the Default Mode Network (DMN) in young binge drinkers along two years. Magnetoencephalography signal during eyes closed resting state as well as Diffusion Tensor Imaging (DTI) were acquired twice within a 2-year interval from 39 undergraduate students (22 controls, 17 binge drinkers) with neither personal nor family history of alcoholism. The group comparison showed that, after maintaining a binge drinking pattern along at least two years, binge drinkers displayed an increased brain connectivity of the DMN in comparison with the control group. On the other hand, the structural connectivity did not show significant differences neither between groups nor over the time. These findings point out that a continued pattern of binge drinking leads to functional alterations in the normal brain maturation process, even before anatomical changes can be detected.

In the last decades, binge drinking (BD) alcohol consumption has taken social relevance given its high prevalence in adolescence^1^,^2^. This consumption pattern is characterized by the intake of large quantities of alcohol in a short interval of time, followed by periods of abstinence^3^. Generally, BD has been defined as the intake of 5 or more drinks (4 or more for females) on one occasion within a 2 hour interval (which corresponds to a blood alcohol concentration (BAC) of around 0.08% or above), at least once in the last two weeks or the last month^3^.

Adolescence is a period of ongoing neurodevelopment that is characterized by ordered changes, such as synaptic pruning and myelination^4^, that lead to functional^5^ and structural^6^ networks maturation. Understanding the neurophysiology consequences of BD during youth is essential since an undeveloped brain is more vulnerable to alcohol-induced damage than adult brain^7^. In this sense, animal studies have pointed out that a BD pattern induces more brain damage in adolescent than in adult rats^8^ as well as a higher degree of cognitive impairment^9^. In human BD population, cognitive deficits have been reported especially regarding executive functions^10^,^11^,^12^. Functional neuroimaging studies demonstrated differences in the BD’s brain dynamics during cognitive task performance^13^,^14^,^15^,^16^. From a structural point of view, alterations were also found white^17^,^18^ and gray matter^19^,^20^ associated with BD.

Despite the growing interest that this pattern of consumption generates in the scientific community, the extent in which BD affects the maturation of the functional and structural networks over time is an issue scarcely studied. Functional connectivity (FC) quantifies the connections between different brain regions based on temporal correlation^21^,^22^. To our knowledge, the only study that has analyzed FC in BDs is a previous study of our research group^23^. In this study we examined FC assessed with magnetoencephalograpy (MEG) during resting state and we found a decrease in alpha band as well as an increase of FC in delta, theta and beta bands in young BDs compared to an aged-matched control group. Currently, the FC studies are focused in the characterization of brain networks, where the default mode network (DMN) is one of the most assessed. This network is highly active during an idle state, when the brain is not involved in an externally imposed goal-directed activity, and it deactivates during task performance. The DMN includes brain regions such as the precuneus, posterior and anterior cingulate, middle prefrontal cortex and the inferior parietal cortex^24^,^25^,^26^. Although the DMN has garnered increasing attention during the last decade, no study has described it in BDs so far.

Alternatively, structural information about brain connectivity can be provided by diffusion tensor image (DTI) as it enables modeling of the white matter connections that support structural networks. Using this technique, weighted connectivity measures estimate the number of tracts connecting two regions and the integrity of anatomical connections respectively. For instance, fractional anisotropy, which is an estimation of white matter integrity, has been shown to be reduced in BD population along several white matter pathways^18^,^27^.

However, although both functional and structural connectivity studies have found differences in BD subjects, there are no follow-up studies showing the progression of the brain connectivity damage caused by BD. Accordingly, the present study is framed as a two years follow-up study and seeks to provide new evidence about how the maintenance of this drinking pattern in mild-long term may affect brain connectivity.

## Results

### Functional Connectivity

**Figure 1:**
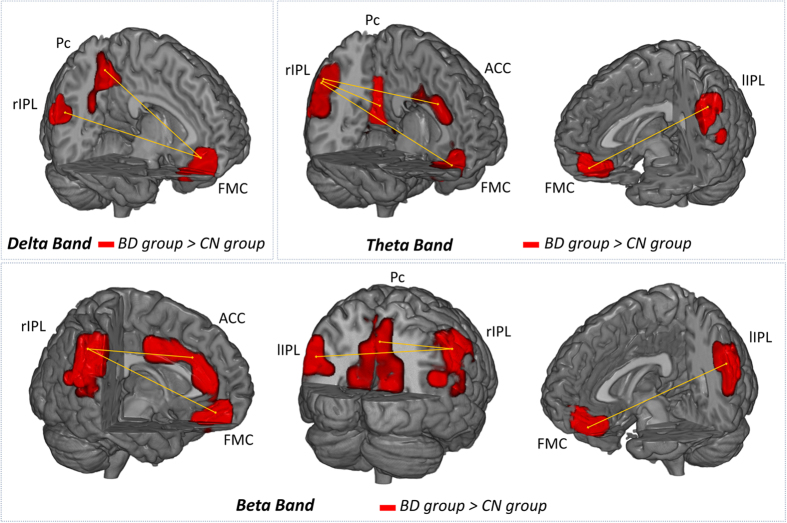
Functional connectivity boxplot. The ratio value (post/pre) of each significant link and group are depicted. Red dotes denote the ratio value of each subject of the binge drinking group (BD) and blue dotes denote the ratio value of each subject of the control group (CN).

**Figure 2:**
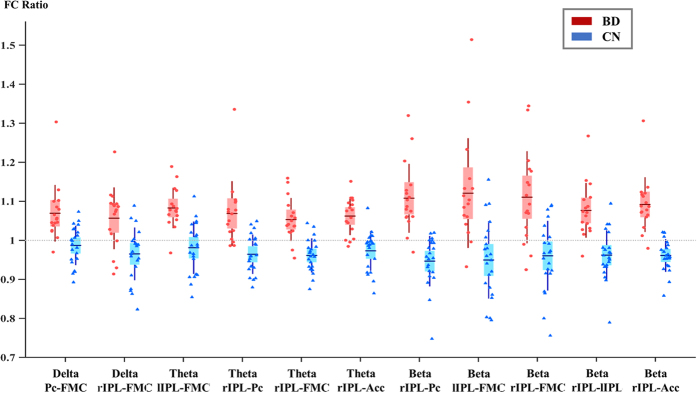
Functional connectivity significant links in delta, theta and beta bands. The ROIs highlighted in red depict the areas of the default mode network with significant (p < 0.05) enhanced FC ratio in the binge drinking group (BD) in comparison to the control group (CN).

Results of the FC analysis are summarized in Table 1 and Figs 1 and 2. We did not found any significant difference between groups in the intra-ROI (intra-Region of Interest) analysis. In the inter-ROI analysis, we found several significant differences (p < 0.05) between groups in the delta, theta and beta frequency bands. In all cases, the BD group showed an increased FC ratio when compared to the CN group. Besides from the group’s comparison, the FC ratios in the BD group were always significantly higher than 1, which implied an increment of the FC along time. By contrast, the CN group showed a stable or decrement FC ratio along time (FC ratio <1 in 8 links, and non-different from 1 in 3 links). Regarding the ANCOVA´s covariate, sex was not significant in any of the connectivity links.

The significant FC network basically pointed out the existence of two kinds of FC patterns: 1) a frontal-parietal pattern, which involved mainly the FMC and its communication with both IPL and the Pc, and 2) a parietal pattern, which consisted of alterations in the communications between the rIPL and most of the DMN ROIs. The frontal-parietal pattern was found in delta, theta and beta frequency bands, whereas the parietal one emerged in theta and beta frequency bands.

Finally, the classification analysis pointed out that it is possible to distinguish between groups with a minimum and maximum of accuracy of 74% and 90% respectively (see Table 2).

### Structural Connectivity

As a result of repeated measures ANCOVA performed with DTI data, we did not find any group differences neither in the pre nor in the post condition. Likewise, no intragroup differences in the BD group were found across time.

## Discussion

This is the first study assessing functional connectivity (FC) along with structural connectivity (SC) in young BD subjects who maintained a pattern of intensive alcohol consumption for more than two years. The results of the present study showed that the FC of the DMN, as assessed with MEG, increased over time in young subjects with a BD pattern compared to the control group. Namely, the BD group showed a significant enhanced FC ratio in several links among DMN ROIs. On those significant ROIs, the FC ratios were always significantly higher than 1 in the BD group, whereas the FC ratios in the control group remained stable or lower than 1. On the other hand, the SC, as assessed by fractional anisotropy, medial diffusivity, radial diffusivity and axial diffusivity did not show significant differences neither between groups nor over time.

To the best of our knowledge, only one study has examined the brain FC in young binge drinkers during resting state. That previous study, carried out by our research group^23^, was conducted when the age of the subjects was 18–19 and we found that the BD group already showed an increased FC in the delta, theta and beta frequency bands in frontal areas, as well as a decreased FC in the alpha band. In the present follow-up study, we have analyzed the rate of change of each group over two years and noticed that the BD group has increased its DMN FC over time in delta, theta and beta frequency bands. By contrast, a decrease of FC of some links of the DMN was observed in the control group. Given the longitudinal nature of the current study, it is important to consider this decrement of FC in the control group in the context of typical adolescent neural maturation. During brain maturation, adolescents exhibit less activation over time, as neural networks become more developed and efficient^28^. As it can be seen in the present results, the typical pattern of neural maturation occur among adolescents who remained nondrinkers, but in the case of young people who continued drinking over those two years, the opposite pattern occurred. These results suggest that alcohol consumption may alter the typical neural development and may produce a developmental delay, hypotheses reported by different studies on binge drinking^20^,^29^,^30^,^31^,^32^.

In this line, this relative brain immaturity might be related to the effects of alcohol on brain receptors. It is well established that moderate to heavy alcohol intake disrupts the normal functioning of brain receptors, mainly N-methyl D-aspartate (NMDA) and gamma-aminobutyric acid-A (GABA-A)^33^,^34^. Given that glutamate-sensitive NMDA receptors has a central role in the synaptic pruning needed to remove and strengthen brain connections^35^, it is possible that a BD pattern during adolescence may interfere with the cortical networks refinement mediated via NMDA receptors. In accordance with this argument, several studies with structural MRI have reported greater grey matter volume in BDs compared to controls in cortical^30^,^31^ and subcortical regions^20^. On the basis of these findings, it has been proposed that this enlarged cortical and subcortical volume would be cause by the reduced pruning resulting from excessive alcohol consumption. Together, these impairments in brain development could explain the opposite pattern of change in the FC of BDs as compared to controls (increase vs. decrease of the DMN FC) observed in the present study.

On the other hand, fMRI studies have also found increased brain activity in the BD subjects during the performance of different cognitive tasks such as verbal learning^36^, working memory^37^ and decision making^13^. This increased brain activity in the BD group, despite having the same behavioral performance, has been interpreted as a compensatory mechanism that allows maintaining an equivalent cognitive performance level. The results of these fMRI studies are no directly comparable with those obtained in the present work, since the BOLD activity obtained with fMRI is different from the FC obtained with MEG, and also the brain networks involved in performing a cognitive task are different from the DMN. However, our results can be added to the existing studies which have found brain hyperactivity in BD population. In a recent study, Wetherill *et al*.^38^ found, in a longitudinal study, that future heavy drinkers showed higher brain activation during response inhibition than nondrinkers after transitioning into heavy drinkers. Similarly, previous EEG studies from our research group showed that BDs, as compared to controls, displayed larger amplitudes in several components of the event-related potentials (which was interpreted as greater neural activity involved in tasks performance), and these differences became to be greater after two years maintaining the BD pattern^14^,^39^. Taken together, these findings are indicating that heavy alcohol consumption may lead to alterations in brain functioning in terms of increasing brain activity both during task performance^38^,^39^ and also during resting state (DMN), as the present study shows.

As mentioned above, in the present study we tested both functional and structural connectivity. Regarding structural networks, we did not find any group differences neither in the pre nor in the post evaluation. Likewise, no intragroup differences in the BD group were found across time. In our view, the brain changes that occur as a result of maintaining BD alcohol consumption are measurable with electrophysiological FC techniques, but maybe the underlying structural changes are not detectable in a structural level.

Finally, the anomalous DMN FC might be considered as a marker of posterior structural and cognitive impairments linked to the maintenance of BD. In this sense, hypersynchronization has been also seen as a biomarker of brain damage in several neurological conditions such as traumatic brain injury^40^ or in early stages of dementia^41^. In a review paper, Bryer *et al*.^42^ developed a model that explains how this brain overactivation reflects brain excitability causing network malfunctioning. In fact, in a sample of mild cognitive impairment patients, higher synchronization was a predictor factor of conversion to Alzheimer disease^43^ and also has been associated with a random network organization^44^.

A number of limitations should be noted; first, the lack of differences between the groups in the anatomical connectivity may be due to the two years’ follow-up time limitation of the study, therefore more longitudinal measures would be needed. Second, although the sample size of the study was considerable for a follow-up study of these characteristics, a larger sample would increase the reliability of the results, and would make possible the assessing of the influence of other factors such as the sex. And finally, the cross-sectional nature of this type of studies makes difficult to draw a conclusion about causal relationship between the physiological differences and the alcohol consumption. So, it cannot be excluded that the differences between groups are previous to the consumption, but in any case, we are able to demonstrate reliable differences of the evolution of the FC between BD group and control subjects.

In conclusion, a continued pattern of BD over at least two years appear to lead to hypersynchronized DMN as compared with the non BD group. This could be taken as a biomarker of potential brain damage caused by alcohol consumption without a clear evidence of deficits on structural connectivity. Understanding the effects of the BD pattern on FC has important implications for the etiology and prevention of future alcohol dependence. Future follow-up studies should explore whether functional networks associated with specific cognitive tasks are as well affected by BD alcohol consumption.

## Methods

### Participants

Thirty-nine undergraduate students of the Complutense University of Madrid (Madrid, Spain) participated in the study. Twenty two were classified as controls (12 females) and 17 as BDs (8 females). The procedure for subject selection is fully described in Correas *et al*.^23^. The participants were evaluated twice within a 2-year interval (at 18–19 and 20–21 years old) and the number of months between evaluations of each group didn’t differ (control group = 22.86 ± 0.89; BD group = 23.26 ± 0.94). The demographic data of each group is shown in Table 2.

Participants were divided into BD and control group according to a questionnaire and a semi-structured interview inquiring about alcohol and other drug consumptions habits. Participants were asked to cover a record of daily consumption indicating what they drank, the quantity and for how long (hours). The BAC was calculated based on the information of the last dinking episode. We considered the BAC as a rough index representing the BD level of each subject. Participants reaching a BAC of 0.08% or above at least once during the last month were classified as BD. On the other hand, the control group consisted of students who never achieved that BAC. Participants were asked to refrain from alcohol consumption for, at least, 24 hours prior to MEG recordings. Subjects were submitted to a breathalyzer test, and the assessment was only performed after verifying a 0.00% breath alcohol level. All volunteers provided written informed consent prior to assessment. The study was approved by the Ethics Committee of the Complutense University of Madrid, Spain and the procedure was performed in accordance with approved guidelines and regulations. Principles of the Declaration of Helsinki were followed.

### MEG Acquisition

Four minutes of MEG signal were acquired (1000 Hz sampling rate and online band pass filter at 0.1–330 Hz) during eyes-closed resting state using a 306-channel (102 magnetometers and 204 gradiometers) system (Elekta©, VectorView). In this study only magnetometers (102 channels) information was submitted to source and statistical analyses. The system was housed in a magnetically shielded room (VacuumSchmelze GmbH, Hanua, Germany). The head movement was monitored by means of four head-position indicator coils attached to the scalp. Ocular movements were tracked with two bipolar electrodes.

### MEG Analysis

#### Preprocessing

The raw recording data were at first submitted to Maxfilter software (v 2.2, Elekta Neuromag) to remove external noise with the temporal extension of the signal space separation method with movement compensation^45^. In this study, we used only magnetometers data in order to avoid mixing MEG sensors with different sensitivities or resorting to scaling. Accordingly, all of the magnetometers’ resting state signals were automatically scanned for ocular, muscle and jump artifacts with Fieldtrip package^46^ and were visually confirmed by a MEG expert. The artifact-free data were segmented in continuous 4 seconds fragments (trials). At least 15 clean trials were obtained from all participants and preserved for further analyses. The number of surviving trials did not differ significantly between groups. To calculate the source´s reconstruction, the time series were filtered in the following frequency bands: delta (2–3.9 Hz), theta (4.1–7.9 Hz) alpha (8.1–11.9 Hz) and beta (12.1–29.9 Hz). The filtering was performed with a finite impulse response filter of order 1500. This filter was applied using a two-pass procedure over the whole four-minute registers, in order to avoid phase distortion and edge effects.

#### Headmodels & Beamforming

A regular grid of 2455 nodes with 1 cm spacing was created in the Montreal Neurological Institute (MNI) template brain. This set of nodes was transformed to each participant´s space using a non-linear normalization between the native T1 image and a standard T1 in MNI template space. The forward model was solved with the realistic single-shell model introduced by Nolte^47^.

Source reconstruction was performed with a Linearly Constrained Minimum Variance Beamformer^48^. For each subject, the covariance matrix was first averaged over all trials to compute the spatial filter´s coefficients and these coefficients were applied to individual trials, obtaining a time series per segment and the source location.

#### Atlas Based Analysis

The FC analysis was performed using atlas-based ROIs. For the subsequent analysis, we specifically focused on FC in the DMN. We set DMN-related ROIs in the precuneus (Pc), posterior cingulate cortex (PCC), anterior cingulate cortex (ACC), frontal medial cortex (FMC) and bilateral inferior parietal lobe (IPL) by referring to the Harvard-Oxford probabilistic^49^. In total, 156 nodes were included in this study as they were located within the 6 ROIs.

#### Functional Connectivity: Phase Locking Value

The FC was measured by means of phase-locking value (PLV)^48^ in each frequency band and was calculated per each trial as explained in^23^. Finally, the results were averaged across trials ending up with symmetrical 156 × 156 nodes connectivity matrices per subjects, phase (pre/post) and frequency band. In order to assess changes over time, we assess the ratio of change between the two phases of the longitudinal study by dividing the value of each connectivity node of the second evaluation between the first evaluation. Finally, to address whether volume conduction could be causing these differences, we have calculated the correlation between beamformer weights in both groups in order to have an estimate of volume conduction^50^. Beamformer weights did not differ between groups in any frequency band, which makes it unlikely that the functional connectivity differences were caused by volume conduction.

#### Statistical Analysis

Clusters of connections, which showed statistically significant group differences (BD subjects vs. control subjects), were explored relying on the cluster-based permutation test^51^ for each frequency band. The methodology was composed by two steps: 1) an intra-ROI analysis that computed the local connectivity within each ROI; and 2) inter ROI FC that evaluated the inter-regional FC among each pair of ROIS of the DMN. In both cases, the procedure was essentially the same. In the intra-ROI analysis, we assessed the FC of all the nodes contained within a ROI; whereas in the inter-ROI the analysis we focused in the FC between the nodes located in the corresponding two bilateral ROIs.

The procedure started by assessing the FC difference between groups for each pair of nodes by means of ANCOVA with sex as a covariate. The significance of the links was assessed using a non-parametric randomization (5000 permutations) testing^52^, Only those links with p-values below 0.05 were kept and included in the following steps of the analysis. Then, we aimed to extract a robust significant network, so-called *network motifs* in graph theory^53^. These networks consisted of several consecutive significant links, which systematically showed a diminished or enhanced FC in the BD group compared with the CN group. We considered a significant motif only when: 1) at least the 25% of the nodes which composed the ROI were involved, 2) at least the 10% of the links among them had significant FC differences between groups, and 3) the motifs should be connected, i.e. there exists a path between each pair of nodes in the motif 54. The first two conditions set the minimum dimensions of the motif, and the third one fixed a constraint in the morphology, dismissing the insulated links. Then, we submitted all the FC values of the links that composed the significant motifs to a cluster-based (in this case motif-based) non-parametric test (5000 permutations)^55^,^56^ to control the multiple comparisons problem. At this point, we wanted to offer a value which would characterize the network of each significant cluster. Thus, we calculate for each significant motif their corresponding degree^53^, i.e. the average PLV-ratio across all links. Then we performed an ANCOVA with sex as covariate between groups, which was corrected by multiplying the *p* value by 5, to further account for the family-wise error for the 5 frequency bands, and we obtained the corresponding effect sizes (Cohen’s d). In addition, we applied a classification analysis using a logistic regression analysis with the leave-one-out cross-validation procedure^43^. Finally, we performed a one-sample t-test to determine whether the value of the ratio of each group differs from a distribution with mean 1.

### MRI Acquisition

MRI was collected from a General Electric 1.5 Tesla using an eight-channel head coil. The imaging protocol consisted of: 3D T1-weighted high-resolution images using a Fast Spoiled Gradient Echo sequence [TR/TE/TI = 11.2/4.2/450 ms; flip-angle = 12**°**; FoV = 250 mm; acquisition matrix = 256 × 256; slice thickness = 1 mm] and Diffusion weighted images (DWI) using a single-shot echo planar imaging sequence [TR/TE = 12000/96.1 ms; FoV = 307 mm; acquisition matrix = 128 × 128; slice thickness = 2.4 mm; NEX = 3]. DWI was acquired along 25 non-coplanar directions with a b-value of 900 s/mm^2^ and 1 image with no diffusion sensitization, i.e. b_0_ image.

### MRI Analysis

#### DWI Analysis

DWIs were corrected for motion and eddy currents using EDDY-FSL, which performs linear affine registration of the volumes to the reference b_0_ volume. The resulting rotations were used to realign the gradient directions matrix. Non-brain tissue was removed from DWI using BET-FSL and diffusion tensor images (DTIs) were obtained using a linear least-squares approach as implemented in FSL-FDT. From that several scalar images representing the shape of the diffusion tensor were obtained: fractional anisotropy, mean diffusivity, radial diffusivity and axial diffusivity.

Rigid transformations were performed between b_0_ and T1-weighted images. In addition, linear affine transformations followed by nonlinear local deformations using FSL-FNIRT were applied to normalize brain-extracted T1-weighted images into the MNI brain template of 1 mm^3^ isotropic resolution. These three transformations were concatenated in one single transformation in order to reduce the number of interpolations, and were applied inversely to transform the JHU white matter tractography atlas^57^ into the diffusion subject-specific space. Average values for the different DTI metrics were obtained for each of the masks included in the JHU atlas.

#### Statistical Analysis

A repeated measures ANCOVA with sex as a covariate was performed with fractional anisotropy, mean diffusivity, radial diffusivity and axial diffusivity data.

## Additional Information

**How to cite this article**: Correas, A. *et al*. Functional and structural brain connectivity of young binge drinkers: a follow-up study. *Sci. Rep.* **6**, 31293; doi: 10.1038/srep31293 (2016).

## Figures and Tables

**Table 1:** Functional connectivity results.

Band/Link	FC Ratio BD group	FC Ratio CN group	Effect Size Cohen’s d	Ancova	Accuracy	Coordinates	Ttest BD	Ttest CN
*Delta/FMC-Pc*	1.07 ± 0.07	0.99 ± 0.05	Δ = 1.7	*p < 2e-04	74%	FMC [−1 43 −16] Pc [9 −59 40]	*p < 1e-03	p < 2e-01
*Delta/FMC-rIPL*	1.06 ± 0.08	0.97 ± 0.07	Δ = 1.4	*p < 5e-04	79%	FMC [−1 45 −15] rIPL [57 −57 23]	*p < 9e-03	*p < 3e-02
*Theta/lIPL-FMC*	1.08 ± 0.05	0.98 ± 0.07	Δ = 1.5	*p < 6e-06	79%	lIPL [−56 −39 40] FMC [−1 43 −17]	*p < 5e-06	p < 2e-01
*Theta/rIPL-FMC*	1.05 ± 0.06	0.96 ± 0.04	Δ = 2.2	*p < 7e-07	87%	rIPL [52 −46 32] FMC [−3 43 −16]	*p < 3e-03	*p < 2e-03
*Theta/rIPL-Acc*	1.06 ± 0.05	0.97 ± 0.05	Δ = 1.8	*p < 3e-06	85%	rIPL [55 −39 34] Acc [2 19 25]	*p < 9e-04	*p < 3e-04
*Theta/rIPL-Pc*	1.07 ± 0.08	0.96 ± 0.05	Δ = 2.1	*p < 3e-05	82%	rIPL [52 −41 38] Pc [−13 −63 20]	*p < 6e-05	*p < 2e-02
*Beta/lIPL-rIPL*	1.08 ± 0.07	0.96 ± 0.06	Δ = 1.8	*p < 5e-06	82%	lIPL [−53 −51 30] rIPL [54 −42 35]	*p < 1e-04	*p < 7e-04
*Beta/lIPL-FMC*	1.12 ± 0.14	0.95 ± 0.10	Δ = 1.7	*p < 6e-05	77%	lIPL [−50 −43 35] FMC [−1 43 −17]	*p < 2e-03	*p < 3e-02
*Beta/rIPL-FMC*	1.11 ± 0.12	0.96 ± 0.09	Δ = 1.7	*p < 6e-05	77%	rIPL [56 −44 30] FMC [0 42 −18]	*p < 1e-03	p < 6e-02
*Beta/rIPL-Acc*	1.09 ± 0.07	0.96 ± 0.04	Δ = 3.2	*p < 2e-08	87%	rIPL [54 −43 33] Acc [0 −19 25]	*p < 3e-04	*p < 9e-03
*Beta/rIPL-Pc*	1.11 ± 0.09	0.95 ± 0.06	Δ = 2.5	*p < 6e-08	90%	rIPL [54 −40 35] Pc [1 −60 35]	*p < 5e-05	*p < 2e-04

The FC ratio was calculated by means of the quotient: post FC/pre FC. ANCOVA test, with sex as covariate, was calculated between groups with the corresponding average FC ratio. The accuracy score was obtained through a logistic regression analysis with the leave-one-out cross-validation procedure. MNI coordinates of the center of each ROI were calculated in the corresponding network. One sample t-test was calculated with the ratio value of each group per each significant link. rIPL/lIPL (right/left inferior parietal lobe). Pc (precuneus). FMC (frontal middle cortex). ACC (anterior cingulate cortex).

**Table 2:** Demographic, tobacco, and alcohol consumption at the first and the second evaluation.

	First evaluation		Second evaluation	
	Controls	Bing Drinkers	Controls	Binge Drinkers
N (females)	22 (12)	17 (8)	—	—
Age	18–19	18–19	20–21	20–21
Handedness (right/left)	22/0	17/0	—	—
Caucasian ethnicity (%)	100	100	—	—
Regular use of tobacco	0	3	0	3
BAC	0.016 ± 0.024	0.166 ± 0.065	0.017 ± 0.029	0.152 ± 0.052

BAC (Blood Alcohol Concentration, grams of alcohol in a BD day, mean ± SD).
